# Ketoanalogue-Supplemented Low-Protein Diet in Patients with Stage 4+ Chronic Kidney Disease in Italy: A Cost–Utility Analysis

**DOI:** 10.3390/nu18071142

**Published:** 2026-04-02

**Authors:** Luca De Nicola, Filippo Aucella, Antonio De Pascalis, Giovanni Stallone, Massimiliano Povero, Linet A. Odonde, Roberta Germanò, Chiara Ruotolo, Maria Serena Russo, Dario Troise, Loreto Gesualdo

**Affiliations:** 1 Nephrologic Unit, Department of Advanced Medical and Surgical Sciences, Università della Campania Luigi Vanvitelli, 80138 Naples, Italy; luca.denicola@unicampania.it (L.D.N.); robertagermano84@gmail.com (R.G.); chiara.ruotolo@yahoo.it (C.R.); 2 Struttura Complessa di Nefrologia e Dialisi, Fondazione Casa Sollievo della Sofferenza, IRCCS, 71013 San Giovanni Rotondo, Italy; f.aucella@operapadrepio.it (F.A.); ms.russo@operapadrepio.it (M.S.R.); 3 UOC Nefrologia e Dialisi, Ospedale VITO FAZZI, 73100 Lecce, Italy; depascalis.a@libero.it; 4 Nephrology, Dialysis and Transplantation Unit, Department of Medical and Surgical Sciences, University of Foggia, 71122 Foggia, Italy; giovanni.stallone@unifg.it (G.S.); dario.troise@unifg.it (D.T.); 5 AdRes, 10155 Turin, Italy; l.odonde@adreshe.com; 6 Unit of Nephrology, Dialysis and Transplantation, Department of Precision and Regenerative Medicine and Ionian Area (DiMePRe-J), University of Bari “Aldo Moro”, 70124 Bari, Italy; loretoge60@gmail.com

**Keywords:** chronic kidney disease, dialysis, cost-effectiveness, low-protein diet, ketoanalogues

## Abstract

**Background/Objectives**: Chronic kidney disease (CKD) is associated with substantial clinical and economic burden, largely driven by progression to dialysis. Nutritional interventions have shown potential in delaying disease progression, yet evidence on their cost-effectiveness remains limited. This study evaluated the long-term cost–utility profile of a low-protein diet supplemented with ketoanalogues (s-LPD) versus a standard low-protein diet (LPD) in patients with stage 4+ CKD from both the Italian National Health System (NHS) and societal perspectives. **Methods**: A Markov model with monthly cycles simulated disease progression from pre-dialysis to dialysis or death. Clinical inputs were derived from the published literature, while costs reflected 2024 Italian tariffs. Three effectiveness scenarios (optimistic, conservative, and pessimistic) were explored to account for uncertainty in the treatment effect. Outcomes included costs, life-years, quality-adjusted life-years (QALYs), and incremental cost–utility ratios. Deterministic and probabilistic sensitivity analyses assessed model robustness. **Results**: Across all scenarios, s-LPD improved survival (up to +0.59 life-years), increased QALYs (up to +0.48), and delayed dialysis initiation (up to +2.88 years) compared with LPD. From the NHS perspective, s-LPD was dominant in the optimistic scenario and cost-effective in both conservative and pessimistic scenarios, with cost savings or only a marginal cost that increases under extreme assumptions. Probabilistic sensitivity analyses confirmed a high probability of cost-effectiveness across scenarios. Results remained robust in additional scenario analyses, including the societal perspective. **Conclusions**: This first Italian cost–utility analysis of s-LPD highlights that s-LPD is a cost-effective strategy for patients with advanced CKD, offering clinically meaningful benefits while reducing or containing healthcare costs. These findings support the adoption of s-LPD as part of conservative management strategies aimed at safely delaying dialysis initiation.

## 1. Introduction

With the rising incidence of kidney failure, chronic kidney disease (CKD) has become the fifth leading cause of death in the World, and its prevalence is predicted to grow by 2040 [1,2,3]. CKD progression imposes substantial economic costs on the healthcare system, largely driven by dialysis treatment in patients who progress to kidney failure [2,4]. The disease burden also derails the quality of life (QoL) of patients, their relatives, and caregivers [3,5]. Thus, measures that delay CKD advancement do benefit patients, as well as the healthcare system and society with regard to CKD-related spending [5].

Over the years, a substantial number of CKD patients have been treated by a low-protein diet (LPD) due to the kidneys’ inability to metabolize protein, which causes toxin build-up in the blood as a result of low glomerular filtration rate (GFR) and increased uremic concentration [4,6]. A very-low-protein diet (VLPD: 0.3–0.4 g/kg/day), with or without ketoanalogues (KAs), has also been proposed for patients with more severe disease [7,8]. However, VLPD may be difficult to sustain in clinical practice, particularly in older CKD patients who have demonstrated low adherence to the regimen by failing to meet their daily protein intake, exposing themselves to malnutrition and sarcopenia [7,8,9].

Today, the Kidney Disease Outcome Quality Initiative (KDOQI) Clinical Practice Guideline for Nutrition in CKD suggests that metabolically stable CKD patients (stages 3–5) not on dialysis be kept on a low-protein diet (LPD: 0.6–0.8 g/kg/day) that will ensure sufficient intake of daily protein without compromising kidney function [7]. These recommendations are aimed at delaying renal failure, reducing mortality, and improving QoL. To achieve even better protein intake and outcomes, a KA-supplemented LPD (s-LPD) is considered. Indeed, KAs do not contain nitrogenous compounds, allowing for their conversion into essential amino acids that support protein synthesis, ensuring protein requirements are met without excreting toxic nitrogen waste into the blood [10]. The most recent Kidney Disease Improving Global Outcomes (KDIGO) Clinical Practice Guidelines also support LPD with targeted supplementation [11].

Several studies have established the clinical impact of s-LPD on pre-dialysis CKD patients. A 2018 randomized controlled trial (RCT) on non-diabetic CKD patients (stage 3b–4) followed for 14 months showed that s-LPD (LPD: 0.6 g/kg/day; KA: 1 tablet/5 kg/day) corrected the fibroblast growth factor 23 (FGF-23) and serum Klotho, which are vital in regulating mineral metabolism to inhibit cardiovascular risk and kidney dysfunction in patients [12]. In another 2024 uncontrolled interventional study, diabetic kidney disease (DKD) patients on s-LPD (LPD: 0.6 g/kg/day; KA: 1 tablet/10 kg/day) demonstrated a 3- and 5-times reduction from baseline in proteinuria and eGFR, respectively, delaying dialysis initiation [13]. Two observational studies on CKD stage 4 patients have also shown the effects of s-LPD, with one study [14] (LPD: 0.6 g/kg/day + KA) demonstrating a 6.8% decline in new cases of end-stage kidney disease (ESKD) needing dialysis initiation, and the other study [15] (LPD: 0.6–0.8 g/kg/day + KA) reported a significantly lower risk of kidney dysfunction (hazard ratio [HR]: 0.13; 95% confidence interval [CI]: 0.09–0.19; *p* < 0.001) and dialysis initiation (HR: 0.24; 95% CI: 0.12–0.49; *p* < 0.001). Similarly, a recent s-LPD meta-analysis revealed a significant delay in CKD progression (*p* = 0.008), particularly in patients with an eGFR > 18 mL/min/1.73 m^2^ (*p* < 0.0001) [16]. The meta-analysis further highlighted no difference in the eGFR of CKD patients on supplemented very LPD (s-VLPD) and s-LPD (*p* = 0.10).

Finally, regarding nutritional status, a recent 2025 cohort study that analyzed 21 elderly patients (90+ years) who were prescribed an s-LPD (LPD: 0.6 g/kg/day; KA: 1 tablet/10 kg/day) as a conservative treatment and followed for 12 months reported no change in the nutritional status of their well-nourished (Subjective Global Assessment score A) patients from baseline, aside from the positive impact on kidney preservation [9].

Previous analyses showed that s-VLPD is a cost-effective alternative to LPD in CKD stage 4+ patients in Italy, Thailand, and Taiwan [17,18,19]. However, evidence on the economic value of s-LPD remains limited. Therefore, this study aimed to evaluate the long-term clinical and economic consequences of supplementing a low-protein diet with ketoanalogues. We conducted a cost–utility analysis comparing KA-supplemented LPD with standard LPD in patients with CKD stage 4+ in Italy.

## 2. Materials and Methods

A Markov model, previously developed to simulate the disease evolution of patients treated with s-VLPD [17], was adapted for the present analysis. The model was updated to evaluate the clinical and cost effects of s-LPD compared with standard LPD given to stage 4+ CKD patients in Italy. LPD was defined as 0.6 g protein/kg ideal body weight/day, while for KA supplementation, a posology of 1 tablet/10 kg ideal body weight/day was assumed according to the study of Piccoli et al. [20]. The monthly cycle model simulated patients at the pre-dialysis phase (estimated glomerular filtration rate—eGFR: <30 mL/min/1.73 m^2^), modeling their outcomes as they advanced to different health states (stayed in pre-dialysis, moved to dialysis, or died). Transition probabilities and costs differed between treatment strategies, while the model structure remained the same as previously described [17].

The analysis was developed from the Italian National Health System (NHS) perspective, accounting for the direct cost of medication, dietary monitoring, and dialysis. A recommended 3% discounting rate was used to model future costs and health outcomes [21]. As scenario analysis, the societal perspective was also considered, by including the indirect costs incurred during illness (productivity loss and the cost of caregivers).

The two alternatives were compared by calculating the incremental cost–utility ratio (ICUR), defined as the ratio between incremental cost (difference between total cost in the s-LPD arm and the total cost in the LPD arm) and the incremental quality of life (difference between total quality-adjusted life years [QALY] in the s-LPD arm and the total QALY in the LPD arm).

### 2.1. Patient Baseline Characteristics

Baseline age and sex reflected the values observed in a cohort of approximately 100,000 CKD patients identified in Italy’s Lazio Region [22]. About 56% of the population evaluated were males, with a mean age of 70 years, whereas the remainder were females with a mean age of 72 years. The average weight input was 74.5 kg, derived from Martino 2024 [8].

### 2.2. Clinical Input

The annual progression from pre-dialysis to dialysis, in patients treated with LPD, was estimated at 24.42% (2.31% monthly) from Garneata 2019 [23]. This value was derived from the 93% cumulative incidence of renal replacement therapy (RRT) over a median follow-up of 114 months reported in the study, assuming a constant hazard over time. For both strategies, the annual mortality risk in pre-dialysis was taken from a prospective study that enrolled 449 patients who followed a moderately restricted LPD in 2007–2015 in the Nephrology Unit of the San Luigi Hospital of Turin [20]. Over a total of 847 person-years of observation, 100 deaths were observed, corresponding to an annual mortality risk of 11.1% (0.98% monthly). The progression from dialysis to death was estimated at 13.8%, resulting from an Italian retrospective observational study from 2008 to 2014 at the Nephrology Unit of Santa Chiara Hospital in Trento, which enrolled 487 patients followed until September 2015 [24].

The treatment effect of s-LPD in delaying dialysis onset was estimated using the adjusted findings (to lower the risk of bias) of two observational studies that explored the impact of LPD supplemented with KA in postponing dialysis. Ariyanopparut 2023 [15] reported a 76% (hazard ratio [HR]: 0.24; 95% confidence interval [CI]: 0.12–0.49) reduction in the risk of dialysis after a median follow-up of 32.9 months. On the other hand, Yen 2022 [14] demonstrated a 38% (HR: 0.62; 95% CI: 0.41–0.94) reduction in the risk of dialysis in year one of treatment. However, the benefit observed in the first year decreased in the long term over the total follow-up of 12 years (HR: 0.91; 95% CI: 0.72–1.14).

Due to the slight inconsistency between the two observational studies, three scenario analyses were performed to confirm the possible benefit despite the selected source of effectiveness. An optimistic scenario was developed using the findings of Ariyanopparut 2023 [15] (HR = 0.24). For the conservative scenario, the HR estimates of Yen 2022 [14] were used (HR = 0.62 in the first 12 months, and HR = 0.91 in the whole follow-up period for the rest of the simulation). The pessimistic scenario was estimated using the same source in the first 12 months (HR = 0.62) and thereafter, no benefit of KA was assumed (i.e., HR was set equal to 1).

### 2.3. Utilities

Specific utility scores were assigned to different health states. Based on the review performed in the previous analysis [17], of the four studies deemed suitable, the utility values of Wyld 2012 [25] were picked as references for the base case since it had utility scores for all the dialysis modes.

### 2.4. Cost Inputs

According to the Italian NHS perspective, only direct healthcare costs were considered in the analysis: drug supplementation (KA and others), dietary monitoring, and dialysis; these costs were sourced from locally available data (2024 ex-factory prices) [26]. The cost of KA (€40.23 per 100-tablet box of Ketosteril) was determined considering a posology of 1 tablet/10 kg of body weight/day derived from Piccoli 2016 [20]. Additional supplements considered were sodium bicarbonate (€39 per 1000-tablet box), vitamin D (€6.32 per 4-tablet box), and calcium (€4.52 per 30-tablet box). Posology and proportion of patients taking each supplementation reflected what was observed in the Garneata [23] trial: sodium bicarbonate in 51% of patients (6.4 tabs/day), vitamin D in 54% (1 tab/week), and calcium in 50% (6.9 tabs/day), for both strategies. Erythropoietin supplementation was not included in the analysis as not reported in the Garneata [23] trial. Diet monitoring cost (€22 per consultation), assumed every two months for each strategy, was estimated from the 2024 Italian outpatient services tariff. For dialysis, the cost per session was estimated by type: hemodialysis (HD) €159.31, continuous ambulatory peritoneal dialysis (CAPD) €55.83, and automatic peritoneal dialysis (APD) €68.25, including yearly catheter maintenance cost (€46.20) and the one-off cost for catheter placement (€179.60) only for peritoneal dialysis (PD) patients. Costs were sourced from the 2024 Italian outpatient services tariff [27]. Using the 2019 Italian census findings of Neri 2022 [28], the proportion of patients on HD and PD was estimated at 83.8% and 16.2% (CAPD 7.7% and APD 8.5%), respectively.

### 2.5. Sensitivity Analyses

A deterministic sensitivity analysis (DSA) was performed to assess the effect of uncertainty in input variables on the outcomes. All variables were varied one at a time while keeping the rest constant. The variation for the costs and dialysis parameters was ±20% of the reference case. The clinical effectiveness and quality of life (QoL) measures were confined within their 95% CIs. The cost-effectiveness was determined by the incremental net monetary benefit (INMB), calculated as the incremental QALYs (QALY_s-LPD_ − QALY_LPD_) multiplied by a pre-specified willingness-to-pay (WTP) threshold, minus the incremental costs (Cost_s-LPD_ − Cost_LPD_). The WTP threshold was assumed equal to €33,000 per QALY as the average ICUR estimated in a recent publication based on 48 positive company submissions to the Italian Medicines Agency (AIFA) [29]. The DSA findings are shown on a tornado diagram, with the large bars at the top representing parameters that contribute the most to the variability of the outcome, hence the point of focus for decision makers.

A probabilistic sensitivity analysis (PSA) was also performed to ascertain the confidence in the findings despite the uncertainty in the input parameters. Samples of input parameters were randomly selected from predetermined distributions used to run the model a thousand times. The results are assessed on a cost-effectiveness plane to get the distribution of outcomes.

### 2.6. Scenario Analyses

Scenario analyses were carried out (for optimistic, conservative and pessimistic scenarios) for the mortality in dialysis, the long-term benefit of KA on mortality, mortality correction for population aging, dialysis starting rule, and the societal perspective.

For mortality in dialysis, an alternative scenario considered 0.290 cases per person-year (annual mortality risk of 25.2%) estimated using the average between the HR = 2.6 for mortality of patients on HD versus those not on dialysis (stage 4+ CKD), and HR = 1.7 for mortality of patients on PD versus those not on dialysis (stage 4+ CKD), which are from findings of a Swedish study [30] conducted on 3040 stage 4+ CKD, 1791 HD, and 725 PD patients.

A long-term benefit of KA on mortality was simulated based on the results of a retrospective study [31] conducted from 2001 to 2013 on patients who received more than 3 months of LPD supplemented with KA in the year preceding the start of dialysis. The study found an all-cause mortality reduction (HR: 0.77, 95% CI: 0.70–0.84) among supplemented patients compared with those on LPD. The evidence of a possible long-term survival benefit of s-LPD is limited and conflicting, but according to the same authors, the benefit expressed seems plausible. Since the mean follow-up was 3.1 years (up to 14 years), the scenario analysis assumed the mortality reduction lasted until death.

The mortality correction for population aging was not performed on mortality in the base case, since the mean age of the modeled cohort was 71 years. However, in the scenario analysis, mortality in each health state was adjusted by a relative risk function depending on the age of patients at each cycle of simulation, estimated from the general population mortality in Italy [32].

The societal perspective was accomplished as an alternative scenario, different from the base case, to account for the indirect costs incurred by the patients and their caregivers due to illness. The cost of the time lost on dialysis was determined by multiplying the monthly paid and unpaid work (household, volunteering, and caring) distributed according to gender and age [33] with the proportion of weekly time lost for each dialysis type: 50% for HD, 30% for CAPD, and 20% for APD, as stated by expert opinion. Caregiver expenses were assigned following the past month’s paid and unpaid work, considering that 63.3% of the caregivers were employed [34], 58% were male, and the mean age was 51.7 years [35]. For each dialysis type, 24.5% of the patients were estimated to require care which would take 2 (PD—assumed to require less support since it is done at night) to 12 (HD—assumed that patients would require 3 hospital visits each week) hours weekly, values obtained from Neri 2022 [28], who reported for PD, and the same value was assumed for HD.

All input data are available in Table A1, Appendix A.

## 3. Results

### 3.1. Cost-Effectiveness Results

In the base case analyses, the optimistic, conservative, and pessimistic scenarios showed that s-LPD led to +0.59, +0.07, and +0.05 enhanced years of survival; +0.48, +0.07, and +0.04 improved QALYs; and approximately €41,000, €3200, and €1200 cost savings, respectively, declaring the treatment dominant over LPD as displayed in Table 1. In addition, s-LPD contributed to +2.88 (optimistic), +0.36 (conservative), and +0.23 (pessimistic) years of delayed dialysis start, offering both clinical and economic benefits over the comparator.

**Figure A1:**
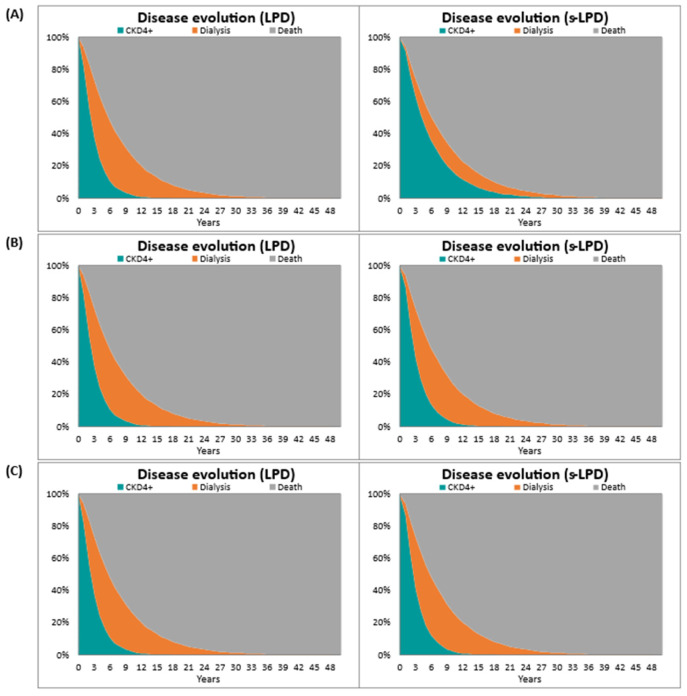
Disease evolution in patients treated with LPD and s-LPD: (**A**) Optimistic scenario; (**B**) conservative scenario; (**C**) pessimistic scenario. Abbreviations: s-LPD, supplemented low-protein diet; LPD, low-protein diet.

Results of the CKD evolution are shown in Figure A1, Appendix A.

### 3.2. Deterministic Sensitivity Analyses

**Figure 1:**
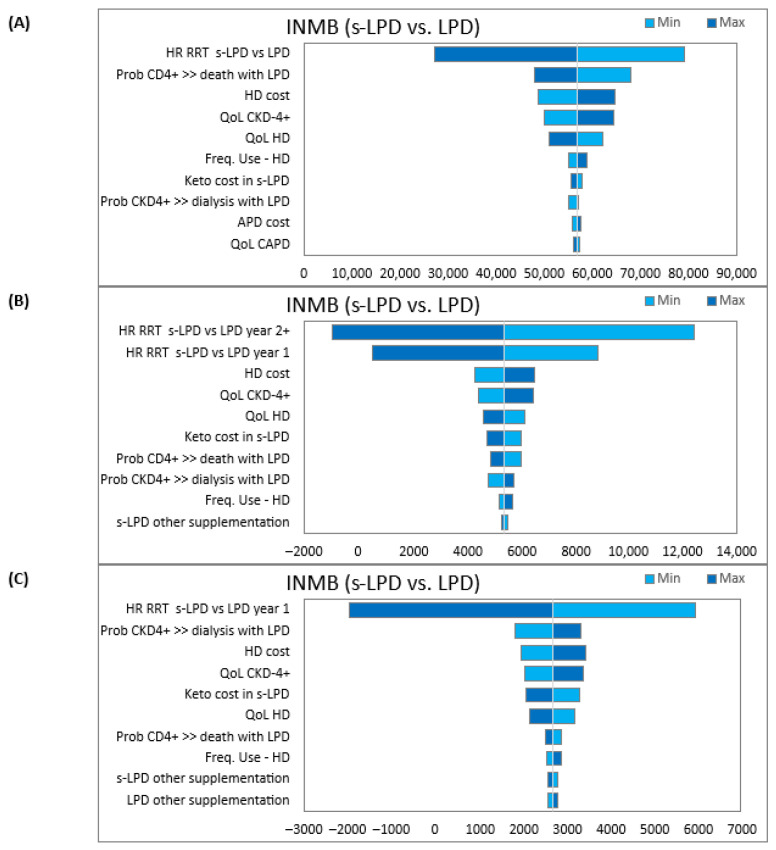
Tornado diagram of incremental costs resulting from DSA: (**A**) Optimistic scenario; (**B**) conservative scenario; (**C**) pessimistic scenario. Abbreviations: DSA, deterministic sensitivity analysis; INMB, incremental net monetary benefit; s-LPD, supplemented low-protein diet; LPD, low-protein diet; HR, hazard ratio; RRT, renal replacement therapy; CKD, chronic kidney disease; HD, hemodialysis; APD, automatic peritoneal dialysis; QoL, quality of life.

In the optimistic scenario, despite applying the most conservative estimates, s-LPD emerged dominant in all simulations (positive INMB), demonstrating cost-effectiveness over LPD (Figure 1A).

For the conservative scenario, the s-LPD strategy was cost-effective for many parameters (Figure 1B). For an extremely high HR of starting dialysis (for subsequent years), the INMB is negative, indicating that s-LPD is not a cost-effective strategy with respect to LPD. However, in this case, s-LPD is also more effective than LPD, with a lifetime cost increase of less than €1500.

The pessimistic scenario exhibited positive INMB for almost all its variables, implying s-LPD’s cost-effectiveness over LPD (Figure 1C). For an extremely high HR of starting dialysis (in year 1), the INMB is negative, indicating that s-LPD is not a cost-effective strategy with respect to LPD. However, in this case, s-LPD is also more effective than LPD with a lifetime cost increase of less than €2200.

### 3.3. Probabilistic Sensitivity Analysis

The PSA findings (shown on scatterplots for 1000 simulations) for the optimistic, conservative, and pessimistic scenarios confirmed the conclusions of the reference case.

**Figure 2:**
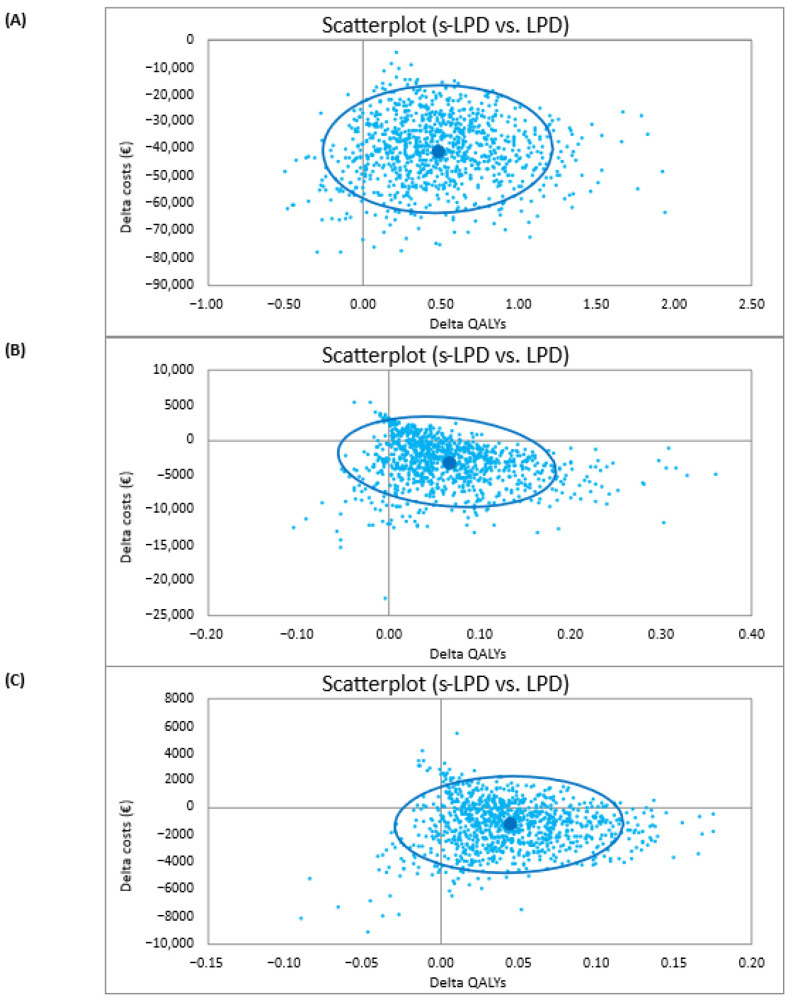
Results of PSA: incremental cost-effectiveness plane: (**A**) Optimistic scenario; (**B**) conservative scenario; (**C**) pessimistic scenario. Abbreviations: PSA, probabilistic sensitivity analysis; s-LPD, supplemented low-protein diet; LPD, low-protein diet; QALYs, quality adjusted life years.

**Figure 3:**
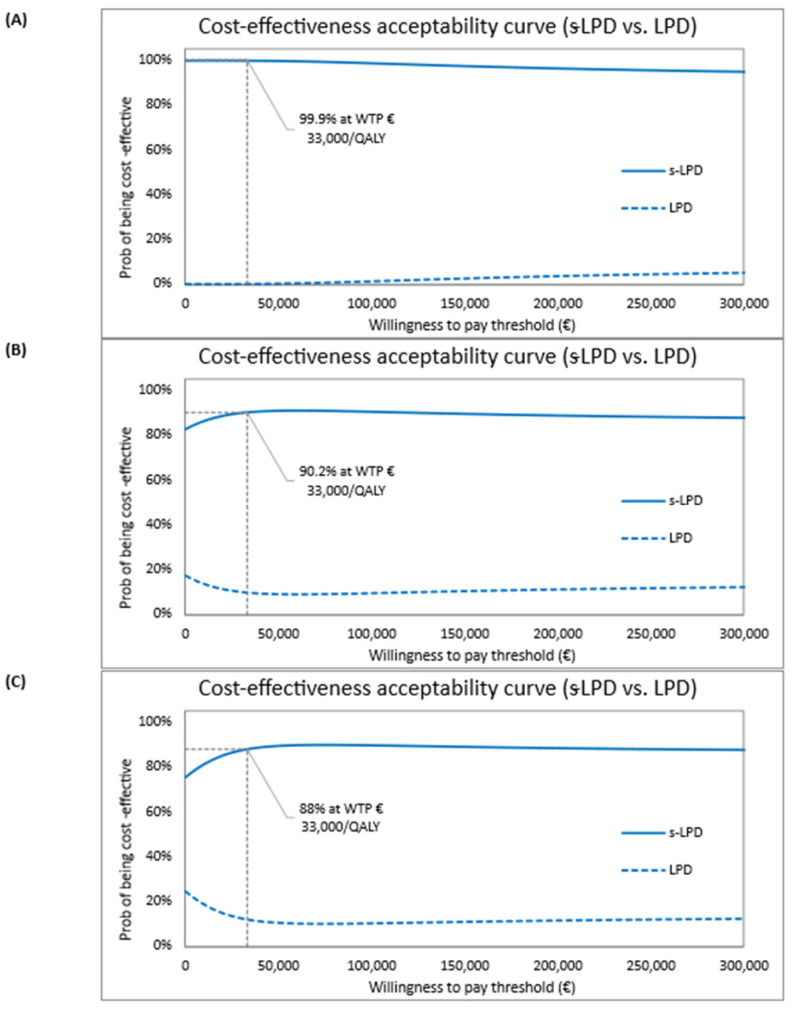
Cost-effectiveness acceptability curve: (**A**) Optimistic scenario; (**B**) Conservative scenario; (**C**) Pessimistic scenario. Abbreviations: s-LPD, supplemented low-protein diet; LPD, low-protein diet; QALYs, quality adjusted life year; WTP, willingness to pay.

Figure 2A shows the optimistic scenario results, indicating that s-LPD amassed €16,000 to €65,000 savings with a QALY gain of −0.26 to 1.21. The ellipse defines nearly a quarter of the area of dominance as 95% probability that CUA is ‘true’. Likewise, the cost-effectiveness acceptability curve (CEAC) shows that s-LPD has almost 100% probability of being cost-effective compared with LPD (Figure 3A).

Figure 2B illustrates the conservative scenario, which shows that s-LPD led to cost savings of −€9400 to €3400 with a QALY gain of 0.06 to 0.19. The ellipse outlines that 95% probability that CUA is ‘true’ falls mostly in the south-eastern area of dominance. The CEAC also confirms that s-LPD has more than 90% probability of being cost-effective compared to LPD (Figure 3B).

Figure 2C displays the pessimistic scenario, which designates −€4800 to €2300 savings with KA use, and a QALY gain of −0.03 to 0.11. In the ellipse, the 95% probability that CUA is ‘true’ falls almost entirely in the north-eastern quadrant of cost-effectiveness and the south-eastern quadrant of dominance. Equally, the CEAC shows that s-LPD has up to 88% probability of demonstrating cost-effectiveness when compared with LPD (Figure 3C).

### 3.4. Further Scenario Analyses

s-LPD was dominant or cost-effective compared to LPD in all scenario analyses in the optimistic, conservative, and pessimistic settings (Table 2).

## 4. Discussion

This analysis investigated the cost-effectiveness of s-LPD vs. LPD among stage 4+ CKD patients in Italy, evaluating for the first time three scenarios (optimistic, conservative, and pessimistic) in the NHS and societal perspectives in Italy. In the absence of large, long-term randomized trials directly comparing s-LPD and LPD, observational evidence currently represents the best available source. Extensive scenario analyses were therefore designed to bracket plausible effect sizes.

The main findings are that s-LPD versus the comparator improved survival and quality of life, delayed dialysis initiation, and reduced the economic impact according to both NHS and societal perspectives.

The sensitivity analyses (DSA and PSA) confirmed the dominance of s-LPD versus LPD in the optimistic scenario, generating savings despite the study using extremely conservative values. For the conservative and pessimistic scenarios, s-LPD was cost-effective for almost all parameters with a negligible increase in lifetime costs only for extremely (maybe unrealistic) values of HR.

Further scenario analyses, including the societal perspective, also established the clinical and cost effectiveness of s-LPD versus LPD.

In general, the present study highlights the potential of s-LPD in terms of clinical and cost benefits. The s-LPD demonstrated the ability to delay the start of dialysis (range: +0.23 to +2.88 years), improve QALYs (range: +0.04 to +0.48), and extend survival (range: +0.05 to +0.59 years). Although s-LPD incurred higher costs for therapy and diet monitoring, these expenses were offset by savings on dialysis in all scenarios. Moreover, the cost-effectiveness profile of s-LPD was qualitatively comparable to that estimated for s-VLPD in the previous analysis [17], suggesting that in more fragile patients or in those unlikely to adhere to a VLPD, an s-LPD strategy may provide similar benefits.

A key strength of the present analysis lies in the comprehensive exploration of uncertainty surrounding the effectiveness of s-LPD through the evaluation of three distinct scenarios—optimistic, conservative, and pessimistic—reflecting a wide range of plausible real-world outcomes. This approach was specifically designed to address the limitations inherent to the available observational evidence and to avoid over-reliance on a single set of assumptions. Notably, across all three scenarios, s-LPD consistently emerged as a cost-effective strategy compared with standard LPD, demonstrating the robustness of the findings even under highly conservative or unfavorable assumptions. This consistency substantially strengthens the credibility and generalizability of the results, supporting the conclusion that s-LPD represents a valuable intervention in advanced CKD irrespective of the magnitude of its clinical benefit. Furthermore, to the best of our knowledge, this study represents the first cost–utility analysis conducted in Italy to specifically assess the long-term clinical and economic impact of s-LPD in stage 4+ CKD patients. By integrating locally relevant cost data with international clinical evidence, the analysis provides novel and context-specific insights for Italian healthcare decision-makers, filling an important gap in the current health economic literature on nutritional interventions in advanced CKD.

The current study findings align with those reported in other settings, including Kazakhstan, Taiwan, and Hungary, which confirmed improved QALYs and savings with s-LPD versus LPD [19,39,40]. The results also indicate the supplement’s potential to delay dialysis in stage 4+ CKD patients, a verdict supported by Chang et al. [41,42], who both concluded that s-LPD delayed CKD progression without affecting the nutritional status of patients. Wu et al. further admitted that an appropriate KA dosage may lessen the risks of ESKD and mortality in anemic advanced CKD patients by slowing the progression of the disease [43]. A 2024 meta-analysis on stage 3–5 CKD patients established the supplement’s ability to postpone dialysis, improve GFR and calcium–phosphate homeostasis without impacting nutritional status and survival [44]. Additionally, Annunziata et al. [9] elaborated that s-LPD was a safe and effective conservative therapy for older patients aged 90+ years with advanced CKD, based on the reduced risk of renal death and kidney failure, improved GFR and azotemia with no influence on the nutritional status. These outcomes, together, reflect the role s-LPD plays in the healthcare system to improve the clinical and economic impact, leading to the sustained well-being of patients. Finally, a recent meta-analysis by Bellizzi et al. [45] has suggested that supplementation of LPD or VLPD with KA may confer renal, metabolic, and nutritional benefits in patients with diabetic kidney disease. Nevertheless, the clinical impact of s-VLPD appears to be influenced by adherence to dietary prescription. Indeed, while substantial benefits of s-VLPD have been demonstrated in the previous randomized trial by Garneata et al. [46], similar effects were not observed in a subsequent long-term pragmatic trial conducted in a broader CKD population [47], in which adherence to both LPD and VLPD was suboptimal (0.83 and 0.6 g/kg/d, respectively). In this context, s-VLPD was safe but did not result in additional benefits compared with a standard LPD, underscoring dietary adherence as a key determinant of its effectiveness in clinical practice.

Nevertheless, some limitations should be considered when interpreting the findings of our study. First, the value used to estimate the mortality of pre-dialysis stage 4+ CKD patients was from an observational study conducted in Italy’s Nephrology Unit of San Luigi Hospital in Turin between 2007 and 2015. The patients studied followed a moderately restricted LPD; not all were at stage 4+. At baseline, the patients’ eGFR was 20 mL/min/1.73 m^2^, with the mortality rate of those with eGFR <15 or <10 mL/min/1.73 m^2^ slightly over that of the entire patient base. All in all, the results show that s-LPD can mitigate disease evolution among patients with more progressed CKD without increasing the mortality risk.

Second, the absence of local clinical data should be considered a limitation. Dialysis onset in LPD patients was estimated using the results of a 15-month RCT (follow-up of 120 months) conducted in a Romanian nephrology center [13]. The population studied was assumed to reflect the Italian situation.

Last, the direct costs (NHS perspective) were largely related to medication, including KA and other supplements (sodium bicarbonate, vitamin D, and calcium), diet monitoring, and dialysis. Indirect costs (societal perspective) accounted for the patient’s productivity loss, transportation, dialysis-related complications, medical supplies, home changes, and caregiver expenses, leaving out unquantifiable aspects. A comprehensive approach to include all costs will shed more light on the cost-effectiveness of s-LPD, demonstrating its capability to lessen costs and the practical burden on patients and their relatives.

Overall, the adoption of healthcare policies for safely delaying dialysis initiation is essential to mitigate the rising prevalence of ESKD and substantially reduce associated costs for patients and the healthcare system.

## 5. Conclusions

This study supports the utility of s-LPD in improving clinical and economic outcomes. The 1000 simulation models, using locally available cost data and clinical data from the available literature, suggest the dominance of s-LPD over LPD, and its ability to provide enhanced clinical outcomes at low cost.

## Figures and Tables

**Table 1:** Base case results.

		Optimistic Scenario		Conservative Scenario		Pessimistic Scenario	
Variable	LPD	s-LPD	Δs-LPD vs. LPD	s-LPD	Δs-LPD vs. LPD	s-LPD	Δs-LPD vs. LPD
**Survival (years)**	**7.27**	**7.87**	**0.59**	**7.35**	**0.07**	**7.32**	**0.05**
Time pre-RRT	2.54	5.42	2.88	2.90	0.36	2.77	0.23
Time in dialysis	4.74	2.45	−2.29	4.45	−0.28	4.55	−0.18
Number of dialysis sessions	902	466	−436	847	−54	867	−35
Number of monitoring visits	15	33	17	17	2	17	1
**QALYs**	**4.45**	**4.94**	**0.48**	**4.52**	**0.07**	**4.50**	**0.04**
**Total costs (€)**	**91,487.95**	**50,699.95**	**−40,829.71**	**88,265.80**	**−3222.15**	**90,252.68**	**−1235.27**
Keto analogous (€)	0.00	5504.63	5504.63	3155.94	3155.94	3031.65	3031.65
Diet monitoring (€)	354.84	702.41	305.85	402.71	47.86	386.85	32.00
Dialysis (€)	90,578.71	43,395.49	−47,183.22	84,077.98	−6500.73	86,229.78	−4348.92
Other supplementation (€)	554.40	1097.43	543.02	629.18	74.78	604.40	50.00

Abbreviations: s-LPD: supplemented low-protein diet; LPD: low-protein diet; QALYs: quality-adjusted life years; RRT: renal replacement therapy.

**Table 2:** Scenario results.

Scenario	Optimistic Scenario			Conservative Scenario			Pessimistic Scenario		
	ΔQALY	ΔCost (€)	ICUR (€/QALY)	ΔQALY	ΔCost (€)	ICUR (€/QALY)	ΔQALY	ΔCost (€)	ICUR (€/QALY)
**Base case**	**0.484**	**−40,788**	**Dominant**	**0.067**	**−3222**	Dominant	**0.045**	**−1235**	**Dominant**
Mortality on dialysis from Neovius 2014 [30]	1.073	−20,146	Dominant	0.148	−380	Dominant	0.099	665	6727
Long-term benefit of s-LPD on mortality	0.774	−30,641	Dominant	0.630	16,499	26,209	0.622	18,993	30,541
Aging correction on mortality	0.289	−27,781	Dominant	0.050	−2835	Dominant	0.035	−1287	Dominant
Dialysis starting rule: after 5 years	0.209	−16,301	Dominant	0.049	−1947	Dominant	0.038	−971	Dominant
Dialysis starting rule: after 10 years	0.378	−16,301	Dominant	0.063	−1947	Dominant	0.044	−971	Dominant
Dialysis starting rule: after 15 years	0.447	−16,301	Dominant	0.066	−1947	Dominant	0.045	−971	Dominant
Daily dose KA: 10 tablets	0.484	−39,412	Dominant	0.067	−2433	Dominant	0.045	−477	Dominant
Daily dose KA: 12 tablets	0.484	−38,036	Dominant	0.067	−1644	Dominant	0.045	281	6289
Societal perspective	0.484	−65,766	Dominant	0.067	−6661	Dominant	0.045	−3535	Dominant
Alternative utilities’ source: from Fletcher 2022 [36]	0.559	−40,788	Dominant	0.077	−3222	Dominant	0.051	−1235	Dominant
Alternative utilities’ source: from Gorodetskaya 2005 [37]	0.588	−40,788	Dominant	0.081	−3222	Dominant	0.054	−1235	Dominant
Alternative utilities’ source: from Sekercioglu 2017 [38]	0.429	−40,788	Dominant	0.059	−3222	Dominant	0.040	−1235	Dominant

Abbreviations: s-LPD, supplemented low-protein diet; LPD, low-protein diet; QALY, quality-adjusted life year; KA, ketoanalogues; ICUR, incremental cost–utility ratio.

## Data Availability

All data generated or analyzed during this study are included in this published article.

## Abbreviations

The following abbreviations are used in this manuscript:

AIFA	Italian Medicine Agency
APD	Automatic peritoneal dialysis
CAPD	Continuous ambulatory peritoneal dialysis
CEAC	Cost-effectiveness acceptability curve
CI	Confidence interval
CKD	Chronic kidney disease
CUA	Cost–utility analysis
DKD	Diabetic kidney disease
DSA	Deterministic sensitivity analysis
eGFR	Estimated glomerular filtration rate
ESKD	End-stage kidney disease
FGF	Fibroblast growth factor
GFR	Glomerular filtration rate
HD	Hemodialysis
HR	Hazard ratio
ICUR	Incremental cost–utility ratio
INMB	Incremental net monetary benefit
KA	Ketoanalogue
KDIGO	Kidney Disease Improving Global Outcomes
KDOQI	Kidney Disease Outcome Quality Initiative
LPD	Low-protein diet
NHS	National Health System
PD	Peritoneal dialysis
PSA	Probabilistic sensitivity analysis
QALY	Quality-adjusted life-years
QoL	Quality of life
RCT	Randomized controlled trials
s-LPD	Low-protein diet supplemented with ketoanalogues
VLPD	Very-low-protein diet
WTP	Willingness to pay

## Appendix A

**Table A1:** Model variable inputs.

Variable	Base Value	SE/95% CI	Distribution Used in PSA	Source
**Baseline characteristics**				
Age (years)	70.9	SE = 10% of mean	Normal	[22]
Gender (% female)	44.2%	SE = 10% of mean	Normal *	[22]
Weight (kg)	74.5	SE = 10% of mean	Normal	[8]
**Clinical input**				
HD freq. of use (HD over HD + PD)	83.8%	SE = 10% of mean	Normal *	[28]
PD freq. of use (PD over HD + PD)	=1 − HD freq.use			
CAPD freq. of use (CAPD over CAPD + APD)	47.3%	SE = 10% of mean	Normal *	[28]
APD freq. of use (APD over APD + CAPD)	=1 − CAPD freq.use			
Annual dialysis onset (LPD)	24.4%	SE = 10% of mean	Normal *	[23]
Annual mortality pre-dialysis	11.1%	SE = 10% of mean	Normal *	[20]
Annual mortality in dialysis	13.8%	SE = 10% of mean	Normal *	[24]
HR dialysis onset: optimistic scenario (s-LPD vs. LPD)	0.24	0.12 to 0.49	Lognormal	[15]
HR dialysis onset: yr1 conservative scenario (s-LPD vs. LPD)	0.62	0.41 to 0.94	Lognormal	[14]
HR dialysis onset: yr2+ conservative scenario (s-LPD vs. LPD)	0.91	0.72 to 1.14	Lognormal	[14]
HR dialysis onset: yr1 pessimistic scenario (s-LPD vs. LPD)	0.62	0.41 to 0.94	Lognormal	[14]
HR dialysis onset: yr2+ pessimistic scenario (s-LPD vs. LPD)	0.91	0.72 to 1.14	Lognormal	[14]
**Utility**				
QoL CKD stage 4+	0.790	0.70 to 0.89	Normal	[25]
QoL HD	0.690	0.59 to 0.80	Normal	[25]
QoL CAPD	0.720	0.60 to 0.85	Normal	[25]
QoL APD	0.800	0.69 to 0.91	Normal	[25]
**Cost input**				
KA (monthly cost)	€97.96	SE = 10% of mean	Normal	[26]
Other supplementation (monthly cost)	€19.53	SE = 10% of mean	Normal	[26]
Diet monitoring (monthly cost)	€11.00	SE = 10% of mean	Normal	[27]
HD (cost per session)	€159.31	SE = 10% of mean	Normal	[27]
CAPD (cost per session) **	€55.83	SE = 10% of mean	Normal	[27]
APD (cost per session) **	€68.25	SE = 10% of mean	Normal	[27]
Catheter placement (one-time cost)	€179.60	SE = 10% of mean	Normal	[27]
Productivity lost (65+ years)	€484.12	SE = 10% of mean	Normal	[33]
Caregiver expenses	€605.49	SE = 10% of mean	Normal	[33]

Abbreviations: APD, automatic peritoneal dialysis; CAPD, continuous ambulatory peritoneal dialysis; CI, confidence interval; CKD, chronic kidney disease; HD, hemodialysis; HR, hazard ratio; PD, peritoneal dialysis; PSA, probabilistic sensitivity analysis; QoL, quality of life; LPD, low-protein diet; s-LPD, supplemented low-protein diet; SE, standard error; freq., frequency, %, percentage; KA, ketoanalogue; yr, year. Notes: * In the PSA, the proportion *p* was normalized using the logit transformation, i.e., logit(*p*) = log(*p*/(1 − *p*)) and then the values of logit(*p*) were sampled from a normal distribution; sampled values were then re-converted into probabilities using the inverse sigmoid function s(x) = exp(x)/(1 + exp(x)). ** Annual catheter maintenance added to the tariff.
